# Frequency-dependent oscillatory neural profiles during imitation

**DOI:** 10.1038/srep45806

**Published:** 2017-04-10

**Authors:** Hisato Sugata, Masayuki Hirata, Yuichi Tamura, Hisao Onishi, Tetsu Goto, Toshihiko Araki, Shiro Yorifuji

**Affiliations:** 1 Department of Neurosurgery, Osaka University Medical School, 2-2 E6 Yamadaoka, Suita, Osaka, 565-0871, Japan; 2 Faculty of Welfare and Health Science, Oita University, 700 Dannoharu, Oita, 870-1192, Japan; 3 Endowed Research Department of Clinical Neuroengineering, Global Center for Medical Engineering and Informatics, Osaka University, Suita, Osaka, Japan; 4 Division of Functional Diagnostic Science, Osaka University Graduate School of Medicine, 1-7 Yamadaka, Suita, Osaka, 565-0871, Japan; 5 Department of Occupational Therapy, Osaka Prefecture University, 3-7-30 Habikino, Habikino, Osaka, 583-8555, Japan

## Abstract

Imitation is a complex process that includes higher-order cognitive and motor function. This process requires an observation-execution matching system that transforms an observed action into an identical movement. Although the low-gamma band is thought to reflect higher cognitive processes, no studies have focused on it. Here, we used magnetoencephalography (MEG) to examine the neural oscillatory changes including the low-gamma band during imitation. Twelve healthy, right-handed participants performed a finger task consisting of four conditions (imitation, execution, observation, and rest). During the imitation and execution conditions, significant event-related desynchronizations (ERDs) were observed at the left frontal, central, and parietal MEG sensors in the alpha, beta, and low-gamma bands. Functional connectivity analysis at the sensor level revealed an imitation-related connectivity between a group of frontal sensors and a group of parietal sensors in the low-gamma band. Furthermore, source reconstruction with synthetic aperture magnetometry showed significant ERDs in the low-gamma band in the left sensorimotor area and the middle frontal gyrus (MFG) during the imitation condition when compared with the other three conditions. Our results suggest that the oscillatory neural activities of the low-gamma band at the sensorimotor area and MFG play an important role in the observation-execution matching system related to imitation.

Imitation is a complex process that includes higher-order cognitive and motor functions in the central nervous system^1^. This process requires the transformation of an observed action into an identical movement performed by the observer, which is called the observation-execution matching system^2^,^3^. Through direct matching of observed and executed behaviors, an individual can directly experience an internal representation of another’s actions, feelings, goals, or intentions^4^. The experience of such direct matching has been suggested to engender and support social-emotional and cognitive development^5^,^6^,^7^, and the dysfunction of this system has been proposed as a neural mechanism explaining the lack of social cognition ability found in autism^8^,^9^,^10^.

The mechanism of imitation is thought to be associated with mirror neurons^11^. Mirror neurons were first identified in the premotor cortex (F5)^12^,^13^ and posterior parietal area^14^ in monkeys. These neurons discharge during the observation and execution of an action. Human neuroimaging studies have also demonstrated such mirroring properties over the temporal lobe, parietal lobule, and frontal areas including the inferior frontal gyrus (IFG) and middle frontal gyrus (MFG) using a variety of imaging techniques such as functional magnetic resonance imaging (fMRI)^1^,^15^,^16^,^17^,^18^,^19^,^20^, positron emission topography (PET)^21^, and magnetoencephalography (MEG)^22^,^23^,^24^,^25^. Of these neuroimaging techniques, MEG has several advantages for analyzing brain activity. First, MEG can record a direct correlate of neural activity, whereas fMRI records hemodynamic changes in the brain induced by neuronal activity. Second, MEG has a higher spatial resolution than electroencephalography (EEG)^26^, such that MEG provides spatial information regarding the region of brain activity with greater accuracy than EEG. Third, MEG has a higher temporal resolution than fMRI and PET. Thus, MEG can detect signal changes in neural oscillations with a spatiotemporal resolution higher than those of other noninvasive neuroimaging techniques.

Recently, a number of MEG studies have revealed the neural mechanisms associated with cognitive processes, such as attention^27^,^28^, memory^29^,^30^,^31^, and reading^32^,^33^, by determining the attenuation of cerebral oscillatory power [known as event-related desynchronization (ERD)]. In particular, oscillatory changes in higher frequency bands (>20 Hz) have been demonstrated to be relevant to higher cognitive processes^32^,^33^,^34^. However, no studies have focused on the neural oscillations of low-gamma ERDs associated with imitation. Several studies have addressed low-frequency bands such as alpha^35^ and beta bands^23^. Considering that neural oscillations in the high-frequency band reflect distinct higher-order cognitive processes, we hypothesized that neural activities associated with imitation indicate specific oscillatory profiles in the low-gamma, alpha, and beta bands.

The objective of the present study was to investigate oscillatory neural profiles associated with imitation not only in the alpha and beta bands but also in the low-gamma band. For this purpose, we used MEG to measure neuromagnetic signals of finger movement during imitation, execution, and observation. To investigate the oscillatory neural profiles during imitation, we performed time-frequency analysis and functional connectivity analysis of MEG data. To evaluate the detailed spatiotemporal distribution of the oscillatory neural activities, we used synthetic aperture magnetometry (SAM), which is a spatial filtering technique based on the nonlinear constrained minimum-variance beamformer.

## Results

### Time-frequency profiles and functional connectivities in sensor space

**Figure 1:**
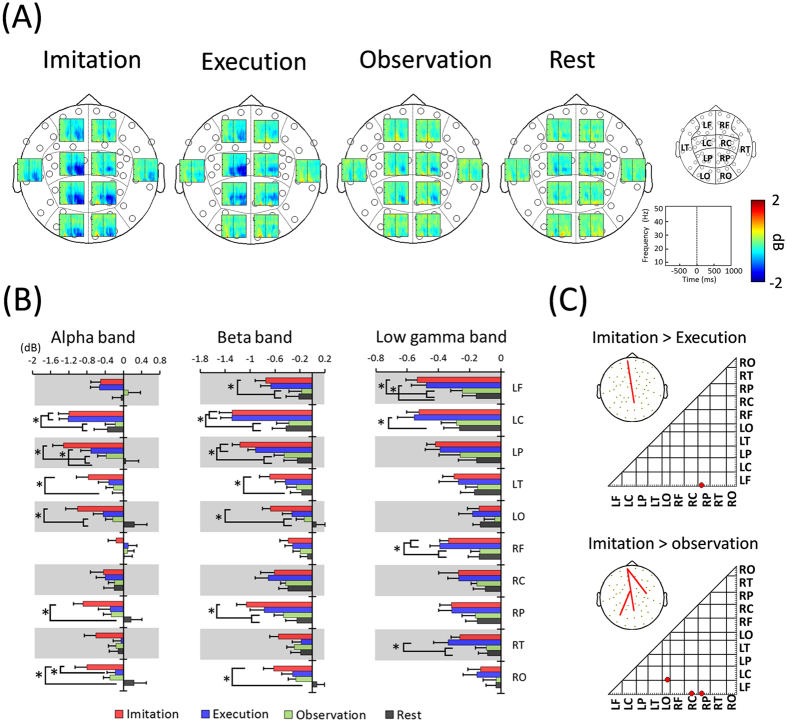
(**A**) Time-frequency maps for each condition. Ten groups of sensors were defined from the MEG sensors (right). Robust ERDs were observed in the alpha, beta, and low-gamma bands at the left central and parietal areas during the imitation and execution conditions. (**B**) ERDs in the alpha, beta, and low-gamma bands were averaged across groups of sensors and time (0–1000 ms). ERDs in the alpha and beta bands in a group of left central sensors during the imitation and execution conditions were significantly lower than those during the observation and rest conditions (*p < 0.05). The beta and low-gamma bands in a group of left frontal sensors showed significant ERDs during the imitation condition compared to the observation and rest conditions (*p < 0.05). Significant differences in ERDs between imitation and execution conditions were observed only in the alpha band in a group of right occipital sensors. Error bars indicate the standard error. (**C**) An imitation-related functional connectivity map in the low-gamma band calculated using IC. When IC during the imitation condition was compared with that during the execution condition (Imitation > Execution), significant IC was observed between a group of frontal sensors and a group of parietal sensors in the low-gamma band (p < 0.05, *FWER*-corrected). When IC during the imitation condition was compared with that during the observation condition (Imitation > Observation), significant IC was observed between a group of frontal sensors and a group of parietal sensors, a group of central sensors and a group of occipital sensors, and a group of frontal sensors and a group of central sensors (p < 0.05, *FWER*-corrected). R = right, L = left, F = frontal, C = central, T = temporal, P = parietal, O = occipital.

In the present study, sensor space analysis was initially performed to examine the time-frequency profiles for each condition from recorded data. Ten groups of sensors were defined from MEG sensors (Fig. 1A right), and power changes at each MEG sensor were averaged across groups of sensors and time (0–1000 ms). The results showed robust ERDs in the alpha, beta, and low-gamma bands in groups of left central and parietal sensors during the imitation and execution conditions (Fig. 1A). In particular, ERDs in the alpha and beta bands in a group of left central sensors during the imitation and execution conditions were significantly lower than those during the observation and rest conditions (Fig. 1B) (alpha band; *F*(3, 44) = 5.36, p = 0.0031, beta band; *F*(3, 44) = 6.42, p = 0.0011, one-way ANOVA). The low-gamma band in a group of left frontal sensors showed significant ERDs during the imitation condition compared to the observation and rest conditions (*F*(3, 44) = 4.66, p = 0.0065, one-way ANOVA). Significant differences in ERDs between the imitation and execution conditions were observed only in the alpha band in a group of right occipital sensors (*F*(3, 44) = 3.875 p = 0.0152, one-way ANOVA).

To investigate the oscillatory neural connectivity, functional connectivity was calculated for each condition using imaginary coherence (IC). To identify the imitation-related connectivity, IC during the imitation condition was compared to those during the execution and observation conditions. The result showed that significant IC was observed only in the gamma band (Fig. 1C). IC was significantly higher for the imitation condition compared with the execution condition between a group of frontal sensors and a group of parietal sensors (t(22) = 3.97, p = 0.0003, family-wise error rate [*FWER*]-corrected). This significant IC was observed even when IC during the imitation condition was compared to that during the observation condition (*t*(22) = 3.68, p = 0.0006, *FWER*-corrected). In addition to this connectivity, significant IC was observed between a group of central sensors and a group of occipital sensors (*t*(22) = 4.43, p = 0.0001, *FWER*-corrected), as well as a group of frontal sensors and a group of central sensors (*t*(22) = 3.89, p = 0.0003, *FWER*-corrected).

### Spatiotemporal distribution of oscillatory neural activities in source space

**Figure 2:**
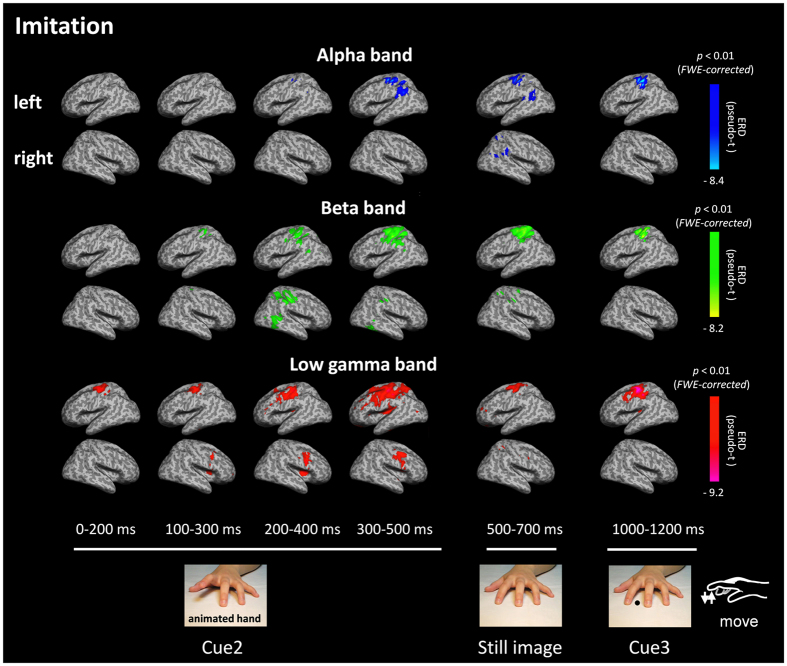
Group statistical maps of spatiotemporal profiles of oscillatory neural activities during the imitation condition. Superimposed images of the alpha (blue), beta (green), and low-gamma (red) bands are shown (p < 0.01, *FWER*-corrected). Significant ERDs in the alpha, beta, and low-gamma bands were observed at he left sensorimotor area during the presentation of Cue 2, when the participants watched the animated hand. Significant ERDs in the low-gamma band were also observed at the bilateral frontal gyri. The majority of the observed oscillatory changes during each condition peaked at 300–500 ms after presenting Cue 2.

**Figure 3:**
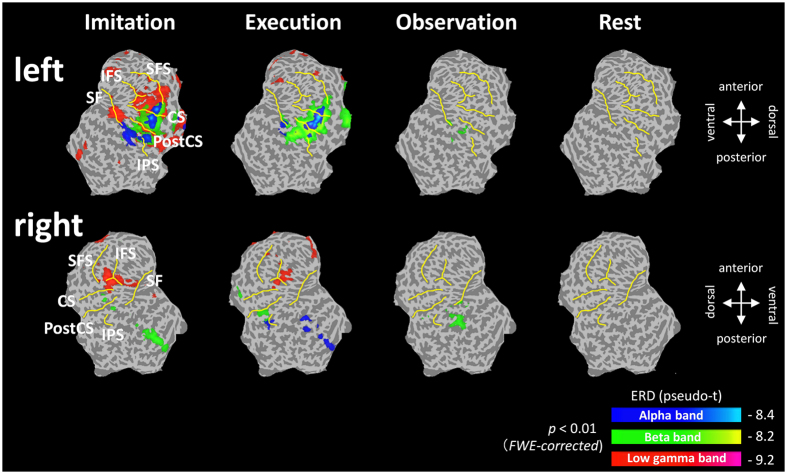
Superimposed results of group statistical maps onto a flattened cortical surface at 300–500 ms after the presentation of Cue 2, during each condition. Significant ERDs were mapped to the flattened surface in the order of blue (alpha band), green (beta band), and red (low-gamma band) (p < 0.01, *FWER*-corrected). Prominent ERDs in the low-gamma band at the sensorimotor and frontal areas were observed only during the imitation condition. SFS, superior frontal sulcus; IFS, inferior frontal sulcus; SF, sylvian fissure; CS, central sulcus; Post CS, postcentral sulcus; IPS, intraparietal sulcus.

**Figure 4:**
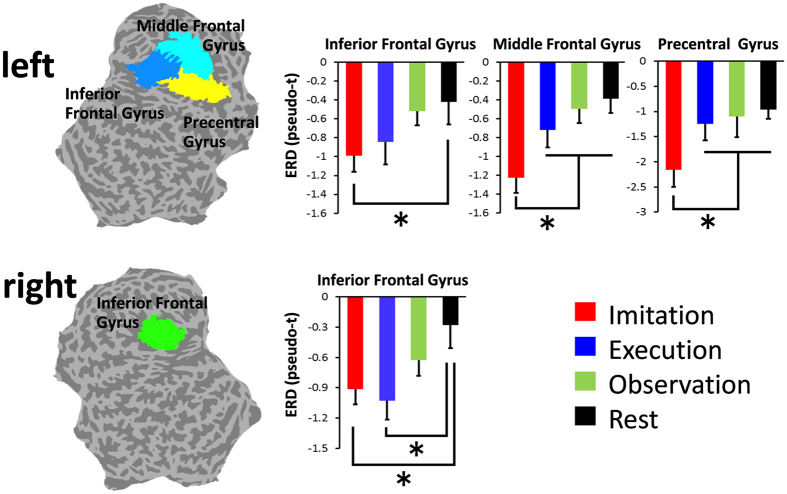
ERDs in the low-gamma band over the frontal areas at 300–500 ms after the presentation of Cue 2. ERDs were spatially averaged over voxels within each ROI. ERDs in the low-gamma band during imitation were significantly lower than those during the other three conditions at the left precentral gyrus and MFG (^*^p < 0.05, ANOVA and Tukey’s method). Low-gamma ERDs at the bilateral IFG differed significantly between the imitation and rest conditions but did not differ significantly between the imitation and execution conditions. Colors on the flattened cortical surface indicate each ROI. Error bars indicate standard error.

In source space analysis, SAM was used to calculate the pseudo-t value for each condition, which is the contrast in source power between the baseline period and the period of interest normalized by sensor noise^36^,^37^. Then, group statistical maps were generated to determine brain regions with significant oscillatory neural activity for each condition. The results showed that significant ERDs in the alpha and beta bands were similarly observed during the imitation and execution conditions at the left sensorimotor area (Figs 2 and S1) during the presentation of Cue 2, in which the participants watched an animated hand during the imitation condition and a static hand during the execution condition. Significant ERDs in the low-gamma band were observed at the left sensorimotor area just after Cue 2 during the imitation and execution conditions. However, these responses were maintained only during the imitation condition and spread to the left frontal gyri (Figs 2 and 3 and Table S1). Significant low-gamma ERDs were also observed in the right inferior/middle frontal gyri at 200–400 ms and 300–500 ms during the imitation and execution conditions (Figs 2, 3 and S1). Majority of these oscillatory changes peaked at 300–500 ms after presenting Cue 2 (Figs 2, 3, S1, S2, and S3) during each condition. Based on these results, ERDs for each frequency band were statistically compared among the conditions at 300–500 ms. The following six regions of interest (ROIs) were anatomically defined using the Montreal Neurological Institute (MNI) template: IFG, MFG, precentral gyrus, postcentral gyrus, inferior parietal lobule, and superior parietal lobule. The results showed that ERDs in the low-gamma band during the imitation condition were significantly lower than those during other conditions at the left precentral gyrus and MFG (*F*(3, 44) = 5.8, p = 0.002, one-way ANOVA) (Figs 4, S4 and S5). In addition, low-gamma ERDs at the bilateral IFG differed significantly between the imitation and rest conditions (left; *F*(3, 44) = 3.47, p = 0.024, right; *F*(3, 44) = 5.01, p = 0.0045, one-way ANOVA), whereas there were no significant differences between the imitation and execution conditions in these regions. Note that the colors on the flattened cortical surface in Fig. 4 indicate each ROI.

## Discussion

Evidence indicates that ERD reflects activated cortical areas^38^ and occurs within a range of motor and sensory paradigms^39^ as well as cognitive paradigms^40^,^41^. In addition, studies showing that the localizations of ERDs, recorded by MEG and those of hemodynamic responses recorded by fMRI are concordant and provide support to the notion that ERDs represent increased neural activation in the cortical area^41^,^42^. Accordingly, our present results using source space analysis indicate that the spatial distributions of specific low-gamma ERDs reflect the brain activity involved in imitation.

From sensor level analysis, robust ERDs were observed in all three frequency bands in groups of left central and parietal sensors during movement tasks. In particular, ERDs in the alpha and beta bands in a group of left central sensors were significantly lower than those during non-movement tasks, suggesting that ERDs in the movement tasks reflect movement preparation^43^,^44^. In contrast, significant differences in ERDs between the imitation and execution conditions were observed only in the alpha band in a group of right occipital sensors. Because participants observed the animated finger during the imitation condition and the static hand during the execution condition, this result may reflect differences in the activation of the visual cortex induced by different types of visual stimuli. Otherwise, no significant differences in ERDs between imitation and execution conditions were observed. These results may be due to the low spatial resolution because time-frequency maps in individual MEG sensors were averaged for groups of sensors.

In the present study, to examine oscillatory neural connectivity related to imitation, sensor level functional connectivity analysis was performed using IC. The results showed that significant IC was observed only in the low-gamma band. When IC during the imitation condition was compared with that during the execution condition, significant IC was observed between a group of frontal sensors and a group of parietal sensors. This significant IC was also observed when compared to that during the observation condition. As axial gradiometers show dipolar topography, sensor level functional connectivity does not represent networks between brain areas directly below them. However, this low-gamma connectivity may partially support the results of previous studies demonstrating the relationship between frontal and parietal areas related to somatomotor transformations and the processing of higher-order visual information^45^,^46^,^47^. In addition to this connectivity, significant IC was observed between a group of central sensors and a group of occipital sensors and between a group of frontal sensors and a group of central sensors when IC during the imitation condition was compared with that during the observation condition. Because imitation requires the transformation of an observed action into an identical movement, it is a more complex central system process than just movement observation. Therefore, IC between a group of central sensors and a group of occipital sensors and between a group of frontal sensors and a group of central sensors in the low-gamma band may also reflect this transformation process (i.e., observation-execution matching system) during imitation.

In addition to the sensor level analysis, we also applied the spatial filtering technique SAM to obtain detailed spatial information on frequency-dependent oscillatory neural activity during imitation. The group statistical maps showed significant ERDs in the alpha and beta bands at the left sensorimotor area when Cue 2 was presented during the movement tasks. This result is consistent with those of previous studies using EEG^48^,^49^, MEG^50^, electrocorticography^51^, and intracerebral recording^52^,^53^,^54^. All these results indicate that these frequency bands are associated with movement preparation. In addition, in the movement tasks, ERDs in the low-gamma band at the right IFG were significantly lower than those in the non-movement tasks during the presentation of Cue 2. Because the right IFG is related to motor imagery or mental simulation induced by an action^55^,^56^, the activity of the right IFG may reflect the mental imaging of finger action. Because participants were required to execute the action after receiving visual stimuli, they may have constructed a mental image of the finger action, forming an inner representation of the same action in the right IFG, even when they only observed a static hand.

During the imitation condition, ERDs in the low-gamma band at the left sensorimotor area and MFG were significantly lower than those during the other three conditions. Several previous studies using EEG and MEG reported that ERDs in alpha and beta bands are localized in the frontal region, including the sensorimotor area, during the observation, execution, and imitation of an action^2^,^3^,^7^,^23^,^57^. Kessler *et al*. (2006) successfully demonstrated the imitation-related functional properties of oscillatory neural activity at 10 ± 4 Hz^35^, suggesting that oscillatory changes in alpha and beta bands play an important role in the observation-execution matching system in humans. In the present study, the group statistical maps showed prominent ERDs in the alpha and beta bands at the fronto-parietal regions, including the sensorimotor area, during the imitation condition, but there was no significant difference when compared with the execution condition. The gap between the results from our study and the previous study^35^ may be due to differences in the experimental paradigm. In a previous study by Kessler, which employed a similar visual stimulus paradigm to that in the present study, participants performed an imitative finger movement as fast as possible in response to the movement stimulus (animated finger), i.e., the observation and action phases were in the same block^35^. In contrast, in the present study, the participants observed the animated finger in Cue 2 (the observation phase) and then imitated the movement after Cue 3 (the action phase), i.e., the observation and action phases were independent. This paradigm might have elicited an enhancement of oscillatory neural activity related to movement preparation in the movement task and therefore decreased the differences between the imitation and execution conditions. In contrast, in the present study, we observed prominent low-gamma ERDs at the left sensorimotor area and MFG only during the imitation condition. As mentioned above, as the observation and action phases were independent in the present study, these low-gamma ERDs may represent neural processes of the movement observation based on imitation. Considering that oscillatory changes in higher frequency bands reflect higher cognitive processes, such as attention, perception, and language processing^32^,^33^,^34^, low-gamma ERDs at the sensorimotor area and MFG may be prominently involved in the imitation. Our results indicate that the low-gamma band at the sensorimotor area and MFG plays an important role in the observation-execution matching system related to imitation.

The present study has several limitations. First, the sample size was small. A sample size of more than 20 subjects and a minimum of 50 trials (ideally > 100) has been recently recommended^58^; in contrast, the sample size of the present study was 12 subjects and 50 trials per condition. Thus, the statistical power of the present study may be weak. Second, this study did not address lower frequency components such as theta bands. A previous study observed no relationship between oscillatory neural activities of theta bands and observation/execution of finger movement. However, a recent study has suggested a relationship between frontal theta oscillations and cognitive control^59^; thus, neural oscillations of the theta band may provide more detailed information related to imitation. Third, defining the strict mapping of cognitive functions by oscillatory neural activity may be difficult because a variety of functions are derived from cortical oscillatory rhythms^60^,^61^. Thus, to reveal the detailed oscillatory neural profiles related to imitation, further study focusing on these frequency components is required.

In summary, this study identified oscillatory neural profiles associated with imitation. The spatial distribution of imitation-specific activities depends on individual frequency bands. In particular, significant low-gamma ERDs are distributed at the left sensorimotor area and MFG. Further, significant connectivities were observed only in the low-gamma band. We assume that low-gamma ERDs at the sensorimotor area and MFG are important for higher-order cognitive and motor processing related to imitation. Thus, we conclude that the oscillatory neural activities of the low-gamma band at the sensorimotor area and MFG play an important role in the observation-execution matching system related to imitation.

## Methods

### Participants

Twelve healthy volunteers (age range, 21–45 years; 4 males and 8 females) participated in this study. All participants were right-handed, which was determined using the Edinburgh Handedness Inventory Test^62^. No participant had a history of neurological or psychiatric disease, and all of them had normal or corrected-to-normal vision. In accordance with the Declaration of Helsinki, we explained the purpose and possible consequences of this study to all participants and obtained their informed consent before the study commenced. The Ethical Review Board of Osaka University Hospital approved this study (No. 11125–5).

### Task

**Figure 5:**
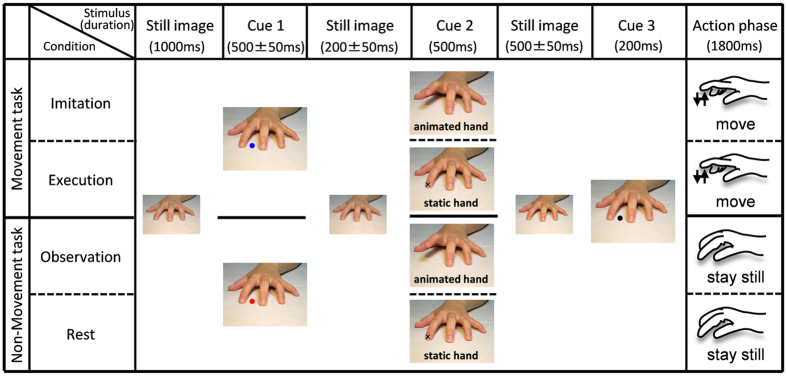
Sequence of images used in the tasks and the schema of the tasks. An epoch started with the presentation of an image of a left static hand (1,000 ms). Then, a blue (indicating movement tasks) or a red dot (indicating non-movement tasks) was presented between the index and middle fingers for the first 500 ms (Cue 1). After presenting Cue 1, two types of visual stimuli were presented for 500 ms (Cue 2) as follows: an animated hand and a static hand. Participants then performed the finger movement in the movement tasks (Blue dot in Cue 1 and animated hand in Cue 2 = imitation condition; Blue dot in Cue 1 and static hand in Cue 2 = execution condition) and did nothing in the non-movement tasks (Red dot in Cue 1 and animated hand in Cue 2 = observation condition; Red dot in Cue 1 and static hand in Cue 2 = rest condition) in response to the presentation of Cue 3.

To investigate the oscillatory neural activity and the connectivities associated with the imitation, we used a modified version of the movement imitation task previously used in fMRI studies^1^,^63^ (Fig. 5). An epoch started with the presentation of an image of a static left hand for 1,000 ms. A Blue or Red dot was then presented between the index and middle fingers for 500 ms (Cue 1). The Blue dot indicated that the trial was a movement task, and the red dot indicated that the trial was a non-movement task. After presenting Cue 1 and the still image (200 ms), two types of visual stimuli were presented for 500 ms (Cue 2) as follows: an animated hand (a single up-and-down movement of the index or middle finger) and a static hand (a cross on the index or middle finger). If an animated hand appeared, the participant observed the movement of that finger. If a static hand appeared, the participant fixed their eyes on the cross on the index or middle finger. After presenting the still image (500 ms) and Cue 3 (black dot, 200 ms), the participants performed the finger movement in the movement tasks and did nothing in the non-movement tasks. Thus, the conditions of this study were as follows:

(1) The Blue dot in Cue 1 and the animated hand in Cue 2: *Imitation condition* The participant observed the animated finger (the movement of the index or middle finger) in Cue 2 and then performed the same movement after Cue 3.

(2) The Blue dot in Cue 1 and the static hand in Cue 2: *Execution condition* The participant observed the static hand with a cross on the index or middle finger in Cue 2 and then performed the finger movement corresponding to the appearance of the cross on the static finger after Cue 3.

(3) The Red dot in Cue 1 and the animated hand in Cue 2: *Observation condition* The participant only observed the animated finger movement in Cue 2 and remained still after Cue 3.

(4) The Red dot in Cue 1 and the static hand in Cue 2: *Rest condition* The participant only observed the static hand with a cross on the index or middle finger in Cue 2 and remained still after Cue 3.

We conducted 50 trials (25 trials each for the index and middle fingers at random) for each of the four conditions, and a total of 200 trials were performed randomly. Note that two types of finger movements were presented to maintain the subject’s concentration, and that static hand images in Cue 1 and Cue 3 and between Cues were presented to enable the participants to prepare the upcoming event (Cue 2 and action phase), respectively. The entire experiment lasted for approximately 20 min.

### Measurements and preprocessing

Measurements were performed with the participant seated in a comfortable chair in a magnetically shielded room. We used a 64-channel whole-head MEG system with third-order SQUID axial gradiometers (NeuroSQUID Model 100, CTF System Ins., Port Coquitlam, Canada). Images of each task were sequentially displayed so that the participant recognized the images as a finger movement. The images were displayed using a visual presentation system (ViSaGe, Cambridge Research Systems Ltd., Rochester, UK) and a DLP projector (Depth Q, MacNaughton Inc., Oregon, USA) on a rear projection screen (RUX87, Kimoto Co., Ltd.) located 1.5 m from participants’ eyes with horizontal and vertical visual angles of 3 degrees and 1 degree, respectively. MEG signals were digitally recorded using an online 200 Hz low-pass filter at a sampling rate of 625 Hz. Notch filters were used at 60, 120, and 180 Hz to eliminate AC line noise. MEG signals were subjected to denoising by independent component analysis (ICA) using fieldtrip (http://www.fieldtriptoolbox.org/). The ICA was based on the infomax ICA algorithm. According to a previous study^64^, we identified eye blinks and motion artifact-related components using the following procedure. (1) We first viewed all ICA activations on a scrolling display and searched for the component with time courses resembling motion artifacts. (2) We verified the nature of candidate components by plotting their scalp topographies, which provided further evidence on their physiological origins. The presentation of Cue 2 was defined as 0 ms, and all time windows were relative to this time. Epochs from −1700 ms to 1300 ms were analyzed.

Anatomical magnetic resonance imaging (MRI) scans were conducted using a 1.5-T or a 3-T (1.5-T or 3-T Signa EXCITE HD, GE Medical Systems) MRI system with a T1-weighted sequence of 130 sagittal slices (1.4-mm thick) with fiducial skin markers at the nasion and bilateral preauricular points.

#### Time-frequency and functional connectivity analyses in sensor space

To delineate the time-frequency profiles for each condition in recorded data, time-frequency analyses were performed for MEG sensor data using fast Fourier transformation (FFT) for each 200-ms time window, with a 25-ms overlap. The baseline was set as the interval between 700 and 900 ms before the presentation of Cue 2, when the participants watched the still hand image, and the analysis period was set at 1000 ms. In accordance with previous studies^32^,^33^, the frequency components were divided into the following three bands: alpha (8–13 Hz), beta (13–25 Hz), and low gamma (25–50 Hz). Ten groups of sensors were defined from MEG sensors (Fig. 1A), and the power changes at each of the MEG sensors were averaged across groups of sensors and time (0–1000 ms). The averaged power changes were then compared between four conditions in each frequency band using ANOVA and Tukey’s method.

To investigate the oscillatory neural connectivity, functional connectivity from 0 to 1000 ms was calculated for the alpha, beta, and low-gamma bands using IC. IC is a connectivity analysis approach that reduces the overestimation biases of EEG/MEG data generated from common references, cross-talk, and volume conduction^65^,^66^,^67^. IC excludes the real parts of coherence that contain similarities with zero time lag and includes the imaginary parts of coherence with similarities to a certain time lag; this is because phase similarities with zero time delay among time series are likely caused by crosstalk or volume conduction. Using only the imaginary parts ensures that the interaction cannot be explained by field spread. Thus, we can evaluate interactions that occur after a certain time lag between sensors in the sensor space analysis^67^,^68^,^69^. IC has recently been described as a promising tool that aims at tackling the effects of field spread prior to performing the inversion step^70^. In the present study, IC of all MEG sensors was calculated using the Fourier transform with a Hanning window in each condition, and the coherence of the alpha, beta, and low-gamma bands was averaged. The connectivity at each frequency band was estimated by averaging across the Fisher’s Z-transformed connections^65^,^66^,^67^. Then, to examine the oscillatory neural connectivity related to imitation, IC during the imitation condition was compared to those during the execution and observation conditions using Student’s *t*-test. In order to control the *FWER*, Bonferroni adjustment was performed for each connection (1953 connections). Sensor pairs with a difference at p < 0.05 corrected by *FWER* were considered statistically significant.

### SAM beamformer analysis

To obtain the frequency-dependent spatiotemporal profiles of imitation with high signal-to-noise ratio and high spatial resolution, the MEG data were analyzed using SAM beamformer (CTF MEG Software, Can), which is a spatial-filtering method that improves the spatial resolution of neuromagnetic sources^41^,^42^. SAM calculates the source power by forming a linear combination of sensors that suppresses the signals from unwanted sources (e.g., noise from the environment and brain) without attenuation caused by the target area. Thus, SAM can distinguish each source generated by multiple areas with high spatial resolution. SAM can also estimate the source power in each voxel from MEG data that have been frequency-band limited by FFT. Estimates of the differences in source power between the baseline period and the period of interest for selected frequency bands and time windows were computed as pseudo-t values^37^. Note that the pseudo-t values are not statistical indexes such as statistical t-values but are the contrast in the source power between the baseline period and the period of interest. In order to increase the resolution of the depth signal, the contrast in the source power was normalized by sensor noise^36^,^37^,^71^. The distribution of pseudo-t values was superimposed on the individual anatomical MR images co-registered to the MEG data.

Here SAM analysis created a volume for covering the entire brain of each individual using a voxel size of 5 × 5 × 5 mm. The baseline period was defined as the interval between 700 and 900 ms before the presentation of Cue 2, and the period of interest was defined as 200-ms time windows sliding by 100 ms starting from 0 to 1300 ms after Cue 2. MEG data were divided into the alpha (8–13 Hz), beta (13–25 Hz), and low-gamma (25–50 Hz) bands by FFT. SAM was then used to compute the covariance of bandpass-filtered MEG data in each time window. Next, SAM estimated the pseudo-t value based on the difference between the source power of the baseline period and that in the period of interest for each frequency band. Group statistical maps were generated to determine the brain regions with significant ERD results in each condition. The statistical significance of ERDs across participants was tested with a nonparametric permutation toolbox for SPM2 called statistical nonparametric mapping (http://warwick.ac.uk/snpm)^72^. The functional images of each subject were normalized using the MNI template^73^. Then, a one-sample t-test was performed at the voxel level using a pseudo-t statistic that incorporated variance smoothing with a 20-mm Gaussian kernel. In order to control the *FWER, FWE*-corrected non-parametric *p* values were calculated using permutation test. The resampling count for the permutation test was 256. Voxels with differences at *p* < 0.01 corrected by *FWER* were considered statistically significant and were superimposed on the template of the inflated cortical surface of the brain extracted by FreeSurfer (http://freesurfer.net/).

After estimating the spatiotemporal profiles of ERDs related to imitation by group statistical analyses, source space ROI analysis was performed using individual pseudo-t values to determine which brain regions/frequency bands contributed to imitation compared with other conditions. Based on the results of the group statistical maps, the following six ROIs were anatomically defined using the MNI template; IFG, MFG, precentral gyrus, postcentral gyrus, inferior parietal lobule, and superior parietal lobule. The individual pseudo-t values were spatially averaged over voxels within each ROI and were compared among the conditions using ANOVA and Tukey’s method.

## Additional Information

**How to cite this article:** Sugata, H. *et al*. Frequency-dependent oscillatory neural profiles during imitation. *Sci. Rep.* **7**, 45806; doi: 10.1038/srep45806 (2017).

**Publisher's note:** Springer Nature remains neutral with regard to jurisdictional claims in published maps and institutional affiliations.

## Supplementary Material

Supplementary Information
