# Supplementary material for: Frequency-dependent oscillatory neural profiles during imitation
Source: Sci Rep. 2017 Apr 10;7:45806. doi: 10.1038/srep45806 (PMC5385530; doi:10.1038/srep45806)
Supplement: Supplementary Information [file srep45806-s1.pdf]

Supplementary Information for

Frequency-dependent oscillatory neural profiles during imitation

Hisato Sugata [1, 2], Masayuki Hirata [1, 3], Yuichi Tamura [4], Hisao Onishi [5], Tetsu Goto [1, 4], Toshihiko Araki [4] and Shiro Yorifuji [4]

[1] Department of Neurosurgery, Osaka University Medical School
2-2 E6 Yamadaoka, Suita, Osaka, 565-0871, Japan

[2] Faculty of Welfare and Health Science, Oita University
700 Dannoharu, Oita, 870-1192, Japan

[3] Endowed Research Department of Clinical Neuroengineering, Global Center for Medical Engineering and Informatics, Osaka University, Suita, Osaka, Japan

[4] Division of Functional Diagnostic Science, Osaka University Graduate School of Medicine
1-7 Yamadaka, Suita, Osaka, 565-0871, Japan

[5] Department of Occupational Therapy, Osaka Prefecture University
3-7-30 Habikino, Habikino, Osaka, 583-8555, Japan

Contents:

Supplementary Figures 1–5

Supplementary Table 1

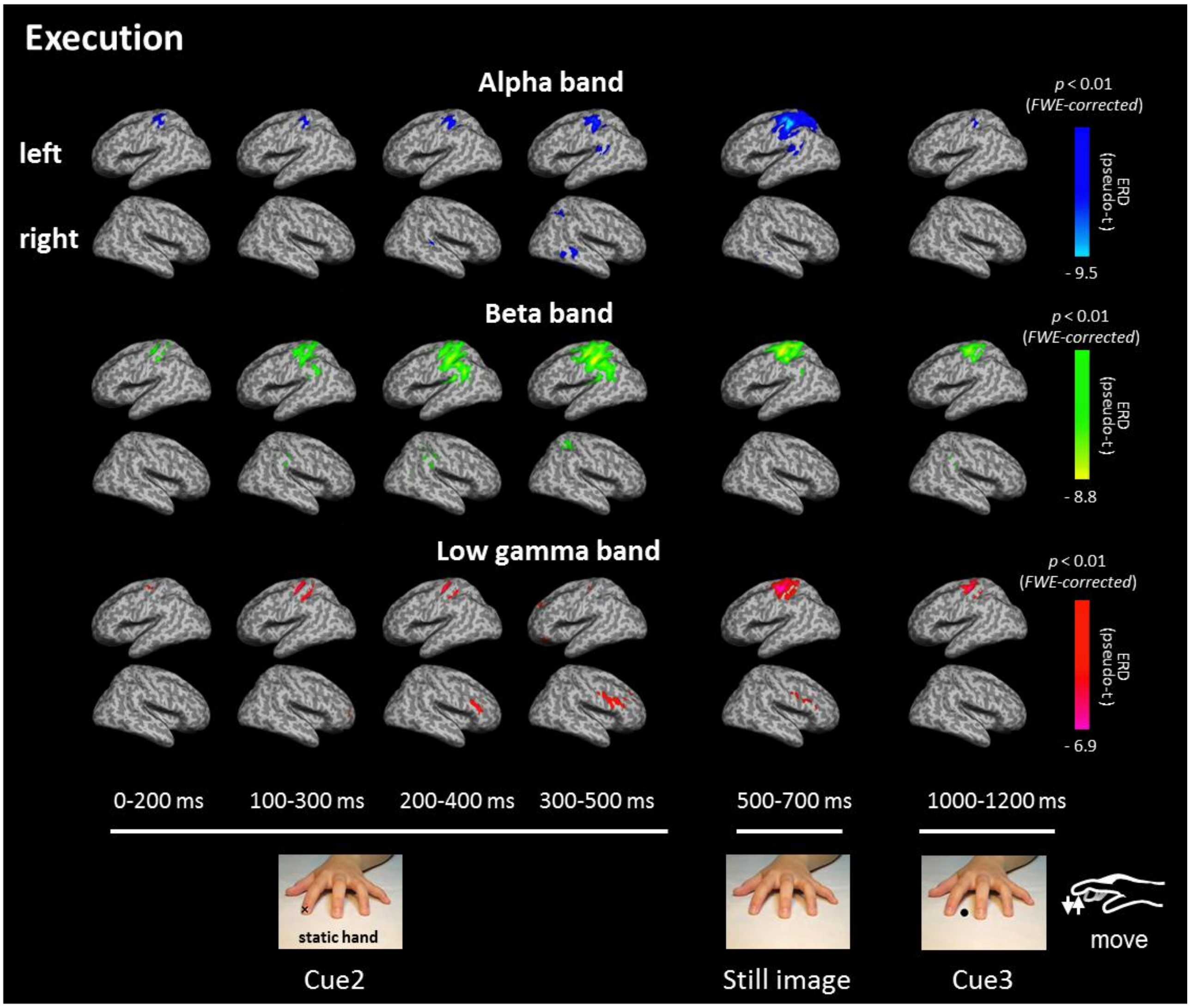

Figure S1. Group statistical maps of spatiotemporal profiles of oscillatory neural activities during the execution condition. The superimposed images in the alpha (Blue), beta (Green), and low-gamma (Red) bands are shown ($p < 0.01$, *FWER*-corrected).

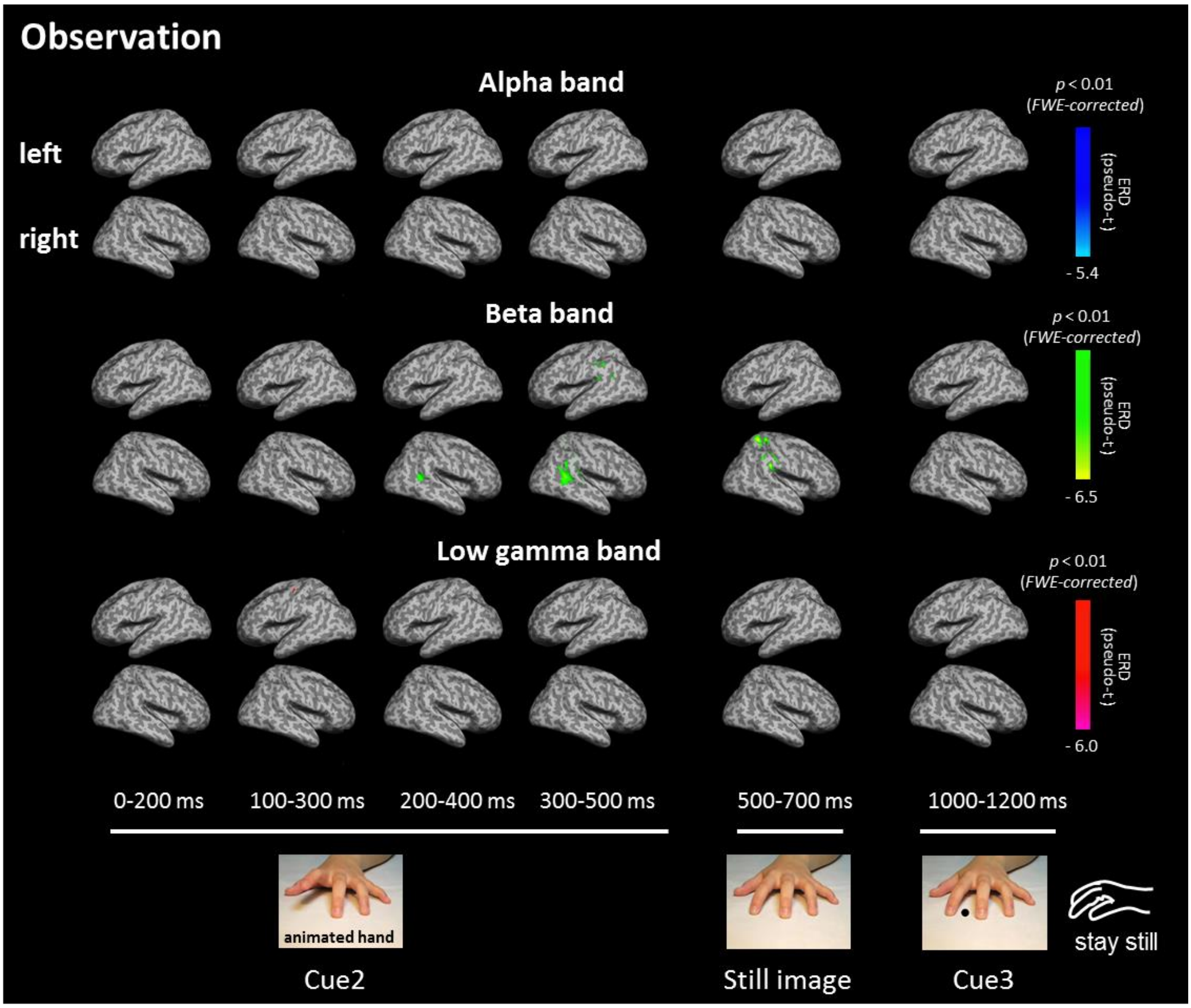

Figure S2. Group statistical maps of spatiotemporal profiles of oscillatory neural activities during the observation condition. The superimposed images in the alpha (Blue), beta (Green), and low-gamma (Red) bands are shown ($p < 0.01$, *FWER*-corrected).

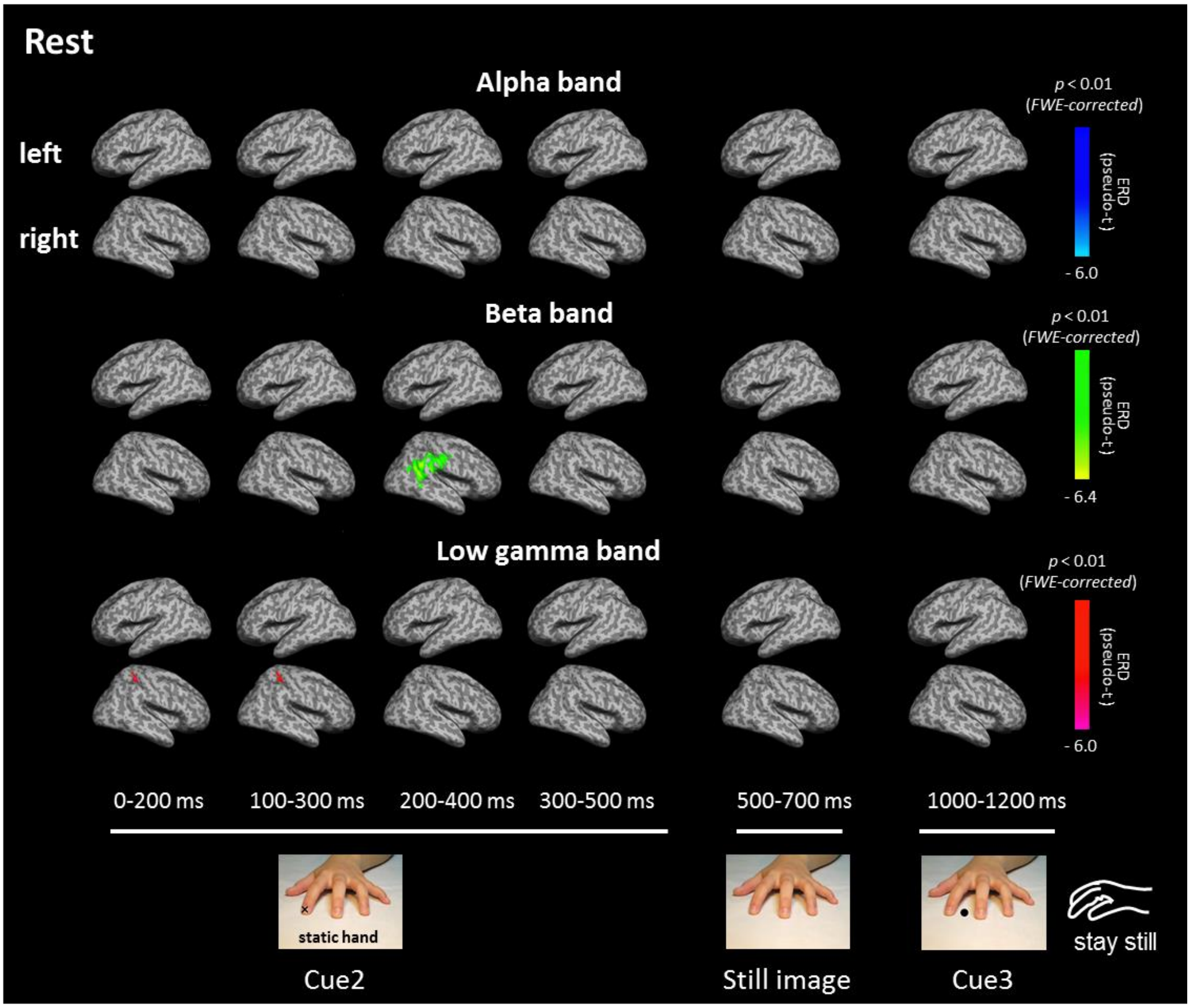

Figure S3. Group statistical maps of spatiotemporal profiles of oscillatory neural activities during the rest condition. The superimposed images in the alpha (Blue), beta (Green), and low-gamma (Red) bands are shown ($p < 0.01$, *FWER*-corrected).

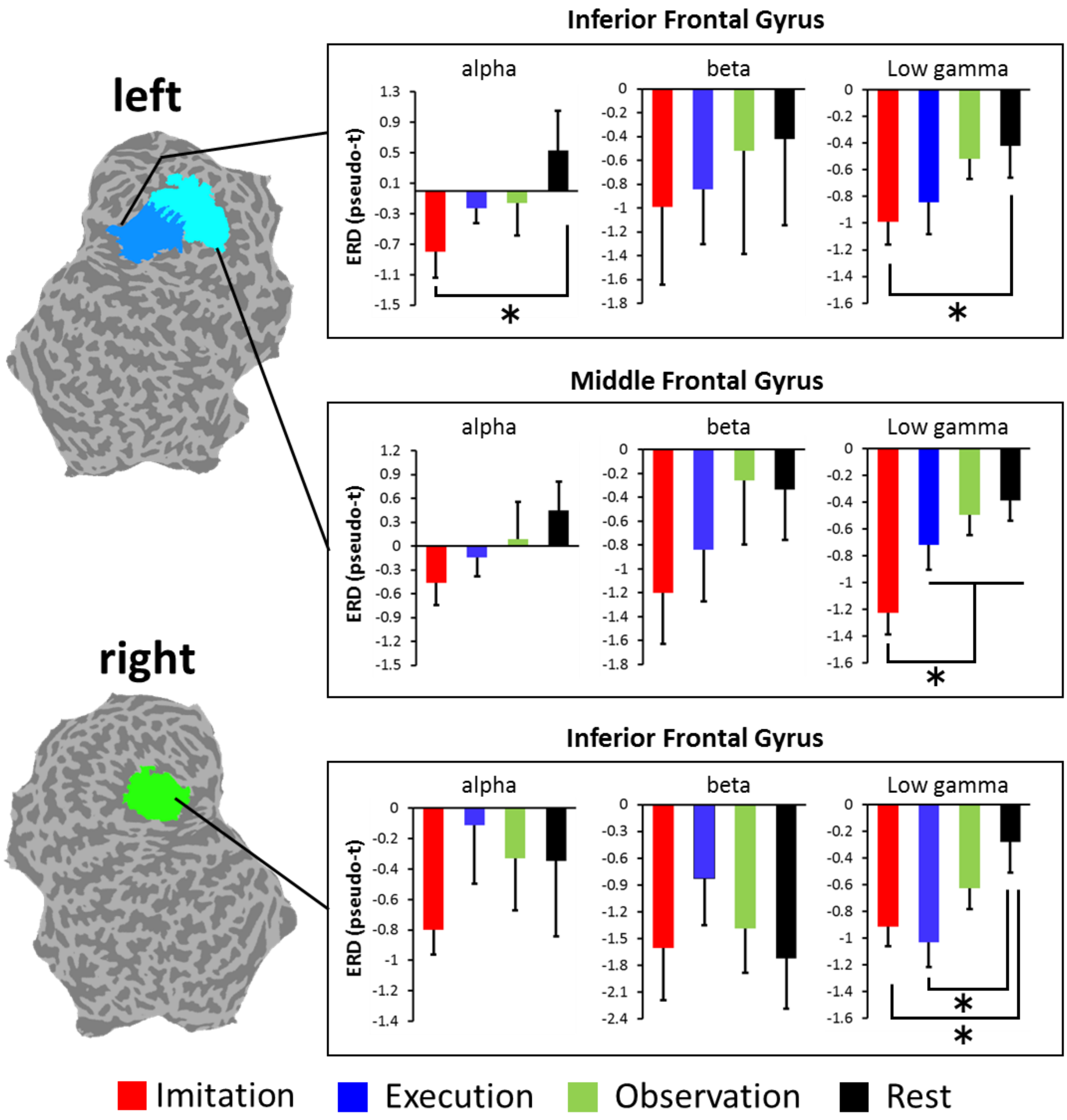

Figure S4. ERDs in frontal areas at 300–500 ms after the presentation of Cue 2. ERDs in the low-gamma band during the imitation condition were significantly lower than those during the other three conditions at the left MFG ($^{*}p < 0.05$). Low-gamma ERDs at the bilateral inferior frontal gyrus differed significantly between the imitation and rest conditions but not between the imitation and execution conditions. Colors on the flattened cortical surface indicate each ROI. Error bars indicate standard deviation.

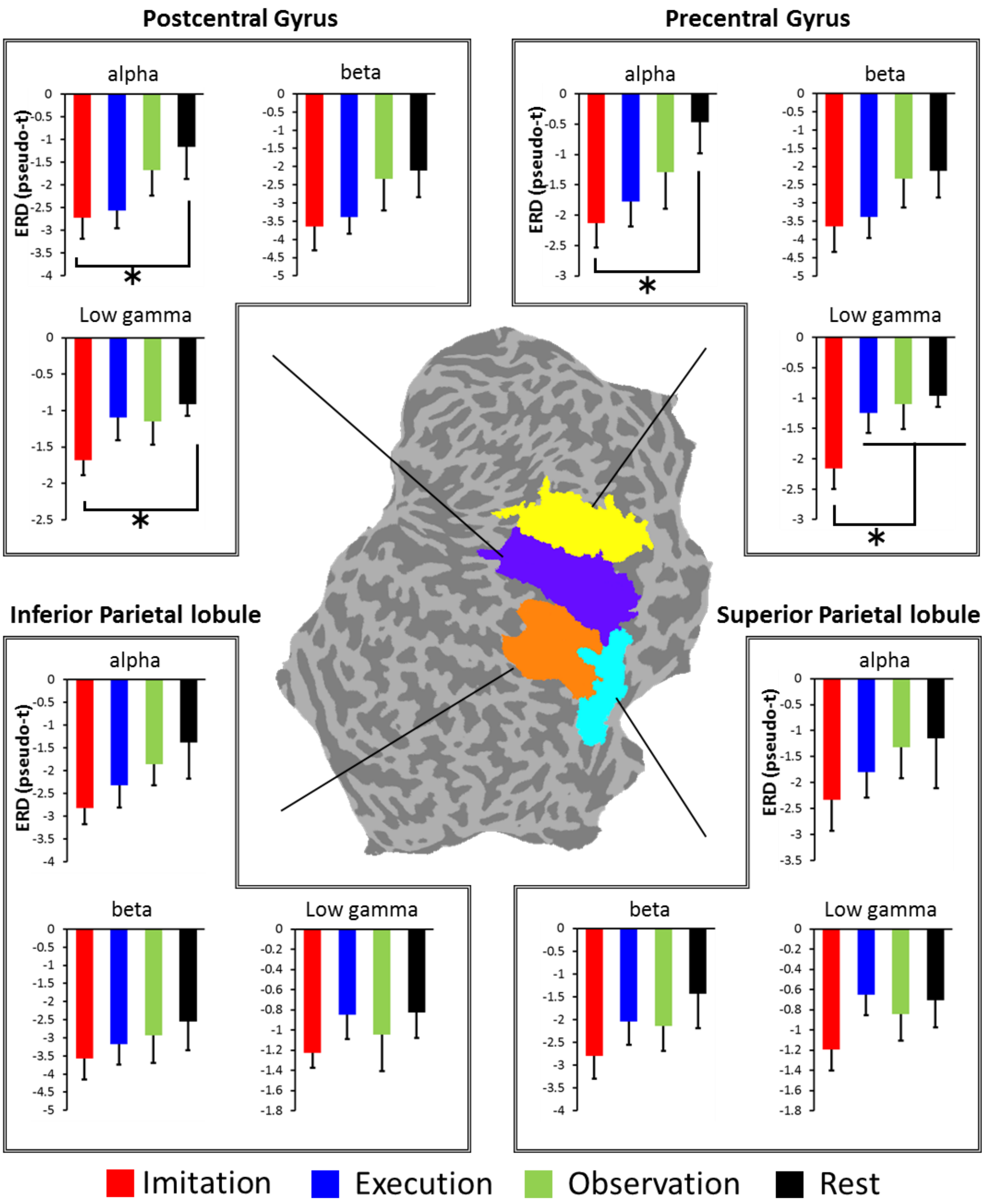

Figure S5. ERDs in the sensorimotor and parietal areas at 300–500 ms after the presentation of Cue 2. ERDs in the low-gamma band during imitation were significantly lower than those during the other conditions at the left precentral gyrus (*$p < 0.05$). The alpha band at the sensorimotor area showed significantly lower ERDs during the imitation condition than that during the rest condition. No significant ERDs were observed in the beta bands over the left frontal, central, and parietal regions between the conditions. Colors on the flattened cortical surface indicate each ROI. Error bars indicate standard deviation.

Table S1. Brain areas showing significant ERDs in each conditions at 300–500 ms

**Frequency band**	**Brain region (BA)**	**Hemisphere**	**MNI coordinates**			**pseudo *t*-value**
			x	***y***	***z***	
			**Imitation**			
<8–13 Hz>	Postcentral Gyrus (3)	Left	−44	−24	56	7.81
	Precentral Gyrus (4)	Left	−44	−20	59	7.06
	Inferior Parietal Lobule (40)	Left	−42	−42	54	6.98
	Inferior Parietal Lobule (40)	Left	−46	−46	30	6.80
	Cingulate Gyrus (24)	Left	−6	−24	42	5.61
	Superior Temporal Gyrus (22)	Left	−58	−39	22	5.55
<13–25 Hz>	Postcentral Gyrus (2)	Left	−46	−28	46	6.99
	Postcentral Gyrus (3)	Left	−42	−24	54	6.72
	Precentral Gyrus (4)	Left	−36	−30	60	6.70
	Fusiform Gyrus (37)	Right	40	−50	−16	6.53
	Middle Occipital Gyrus (19)	Right	48	−56	−11	6.50
	Postcentral Gyrus (3)	Right	50	−26	42	5.74
	Inferior Parietal Lobule (40)	Left	−54	−34	30	5.63
	Superior Temporal Gyrus (22)	Right	66	−48	6	5.43
	Superior Temporal Gyrus (39)	Left	−54	−56	14	5.37
	Precentral Gyrus (6)	Right	34	−12	62	5.18
<25–50 Hz>	Postcentral Gyrus (3)	Left	−40	−22	48	7.77
	Precentral Gyrus (6)	Right	38	4	38	6.56
	Inferior Frontal Gyrus (44)	Left	−47	3	31	6.29
	Precentral Gyrus (6)	Left	−46	−2	34	6.52
	Inferior Frontal Gyrus (44)	Right	44	2	28	6.27
	Middle Frontal Gyrus (8)	Left	−26	10	42	6.25
	Inferior Frontal Gyrus (44)	Right	36	9	40	6.22
	Precuneus (7)	Left	−14	−54	48	6.17
	Superior Parietal Lobule (7)	Left	−28	−60	58	6.06
	Inferior Parietal Lobule (40)	Left	−40	−50	50	5.92
	Insula (13)	Right	46	9	16	5.78
	Middle Frontal Gyrus (9)	Left	−34	24	29	5.72
	Medial Frontal Gyrus (9)	Right	8	44	32	5.54
	Inferior Frontal Gyrus (47)	Left	−24	34	−2	5.38
	Inferior Frontal Gyrus (44)	Left	−24	34	−2	5.38

**Execution**						
<8–13 Hz>	Postcentral Gyrus (40)	Left	−44	−32	52	7.88
	Fusiform Gyrus (37)	Right	34	−46	−12	5.75
	Insula (13)	Left	−46	−40	18	5.74
	Inferior Parietal Lobule (40)	Right	32	−52	42	5.61
<13–25 Hz>	Postcentral Gyrus (2)	Left	−40	−26	46	8.37
	Precentral Gyrus (4)	Left	−38	−17	52	6.59
	Precuneus (7)	Right	31	−46	48	5.36
	Inferior Parietal Lobule (40)	Left	−58	−54	40	5.26
	Medial Frontal Gyrus (6)	Right	2	−26	62	5.24
<25–50 Hz>	Inferior Frontal Gyrus (44)	Right	44	8	25	5.79
	Inferior Frontal Gyrus (47)	Left	−38	30	−18	5.50
	Anterior Cingulate (24)	Right	12	26	20	5.45
	Cingulate Gyrus (24)	Left	−4	−12	38	5.38
	Medial Frontal Gyrus (9)	Right	28	36	24	5.31
	Precentral Gyrus (9)	Left	−38	−28	56	5.25
	Anterior Cingulate (32)	Right	0	26	−10	5.24
	Medial Frontal Gyrus (9)	Left	−20	38	30	5.00
	Precentral Gyrus (4)	Left	−40	−18	58	4.99
**Observation**						
<13–25 Hz>	Insula (13)	Left	−44	−40	24	5.29
	Superior Temporal Gyrus (22)	Left	−58	−60	18	5.05
	Middle Temporal Gyrus (21)	Right	60	−58	2	5.04
	Inferior Parietal Lobule (40)	Right	40	−44	58	5.03
	Postcentral Gyrus (2)	Left	−52	−26	40	4.95
	Superior Temporal Gyrus (22)	Right	64	−24	6	4.91
	Postcentral Gyrus (2)	Right	50	−34	36	4.91
	Inferior Parietal Lobule (40)	Right	62	−52	42	4.83
**Rest**						
	There were no significant ERDs.					

BA: Broadmann's area
